# Additive Manufacturing of Biomedical Constructs with Biomimetic Structural Organizations

**DOI:** 10.3390/ma9110909

**Published:** 2016-11-09

**Authors:** Xiao Li, Jiankang He, Weijie Zhang, Nan Jiang, Dichen Li

**Affiliations:** 1 State Key Laboratory for Manufacturing Systems Engineering, Xi’an Jiaotong University, Xi’an 710049, China; xiaobin.hz@gmail.com (X.L.); jn8754@stu.xjtu.edu.cn (N.J.); dcli@mail.xjtu.edu.cn (D.L.); 2 Department of Mechanical Engineering, McGill University, 817 Sherbrooke Street West, Montreal, QC H3A 0C3, Canada; 3 Department of Knee Joint Surgery, Hong Hui Hospital, Health Science Center, Xi’an Jiaotong University, Xi’an 710054, China; zitengbiyue@163.com

**Keywords:** additive manufacturing, three-dimensional printing, biomimetics, biological model, tissue engineering, vasculature, gradient interface, multicellular system

## Abstract

Additive manufacturing (AM), sometimes called three-dimensional (3D) printing, has attracted a lot of research interest and is presenting unprecedented opportunities in biomedical fields, because this technology enables the fabrication of biomedical constructs with great freedom and in high precision. An important strategy in AM of biomedical constructs is to mimic the structural organizations of natural biological organisms. This can be done by directly depositing cells and biomaterials, depositing biomaterial structures before seeding cells, or fabricating molds before casting biomaterials and cells. This review organizes the research advances of AM-based biomimetic biomedical constructs into three major directions: 3D constructs that mimic tubular and branched networks of vasculatures; 3D constructs that contains gradient interfaces between different tissues; and 3D constructs that have different cells positioned to create multicellular systems. Other recent advances are also highlighted, regarding the applications of AM for organs-on-chips, AM-based micro/nanostructures, and functional nanomaterials. Under this theme, multiple aspects of AM including imaging/characterization, material selection, design, and printing techniques are discussed. The outlook at the end of this review points out several possible research directions for the future.

## 1. Introduction

Additive manufacturing (AM) comprises different technological approaches to fabricate three dimensional (3D) constructs in an additive layer-by-layer manner without the need for a mold. It is presenting unprecedented possibilities for biomedical studies [1]. This technology is particularly good at direct fabrication of complex architectures and compositions, where chemicals, biomaterials, and cells are positioned in a layer-by-layer fashion. Thus it has great potential to replicate the structures and functions of native tissues and organs. Through the rapid advancements in this field over the past decades, researchers have invented a number of printing techniques, explored many material compositions, and created various 3D biomedical constructs with increasing precision and complexity [2,3,4,5].

A typical AM process of biomedical constructs involves four phases: imaging/characterization, design, material selection (e.g., cells, biomaterials, and chemicals), and fabrication. The imaging/characterization phase utilizes tools like micro computed tomography (µCT) and magnetic resonance imaging (MRI) to grasp the structural organization of a target biological system. Characterizations like mechanical measurement also offer insights on the properties of native tissues/organs, to guide the design of AM-based biomedical constructs [6]. In the design phase, deliberation is required in deciding what level of details should be replicated in the AM-based construct and what structural features of the target biological system are the foundations of the biological phenomena of interest. For material selection, the designers need to examine the requirements from two sides: the biomedical application of the AM-based construct demands that the cell and other materials function properly with less biocompatibility and toxicity issues; the materials should be deposited with acceptable efficiency and cell viability using the 3D printer. A broad variety of biomaterials that are suitable for AM has been covered by a number of recent review articles and is thus not a focus of the present article [7,8,9]. There are different techniques (e.g., inkjet bioprinting, laser-aided bioprinting, and micro extrusion) for the fabrication phase, and readers may refer to a review that provides a table to compare the parameters of different techniques, to meet specific demands [10].

AM-based biomedical constructs have their major impact on two clusters of applications. The first cluster serves tissue engineering, where the ultimate goal is to replace or repair dysfunctional organs with implanted biomedical constructs [11]. Compared with conventional techniques that seed cells in porous scaffolds or precursor materials, AM enables a controllable arrangement of biomaterials and/or cells consistent with natural tissues and organs. With improved understanding of the correlation between structures and functions, researchers may be able to produce AM-based tissues and organs that are better substitutes to the natural ones. In the case of implantation, the materials to be deposited or printed could be synthetic or natural, and must have high biocompatibility, proper degradability, and other chemical/physical properties relevant to the environment in the human body. The second cluster of applications is to build tissue/organ models, for such purposes as biological studies, drug screening, and toxicity analysis [12]. It is well accepted that 3D cell cultures provide better biological models than conventional two-dimensional (2D) cell cultures, because cells are more likely to maintain their morphologies and functions in the 3D environment analogous to biological organisms [13]. AM moved forward by adding predefined cell positioning, material composition, and extracellular microenvironment to the systems, empowering researchers to interrogate the complex but controllable models.

A central theme in AM of biomedical constructs is the strategy of designing and constructing biomedical structures. One strategy learns from developmental biology to deposit cells and materials as precursors, and let them develop into a proper organization with corresponding functions [14]. This requires deep understanding of the organogenesis and the mechanisms that drive the biological developmental process. Another strategy, which is widely adopted, is inspired by the structural organizations of the biological organisms. AM is particularly suitable for this strategy, because this fabrication technology has been refining our ability to mimic the details of biomedical structures. Guided by the biomimetic strategy, many achievements have been made for AM of biomedical constructs. Here, we present this review to discuss the progress in several aspects of AM-based biomimetic structural organizations: vasculatures, gradient interfaces, multicellular constructs, and other emerging trends. In order to better cover the existing techniques to fabricate biomedical constructs based on AM, we review the reports where cells and biomaterials were directly deposited, where biomaterials were deposited before cell seeding, or where molds were fabricated before casting biomaterials and cells. We think these topics represent the exciting advances of AM-based biomedical constructs inspired by biology thus far. We also point out possible future directions at the end of the review.

## 2. AM-Based Vascular Constructs

### 2.1. Understanding the Challenge of Vascularization

Only a few thin engineered tissues (e.g., skin) can have their cells supplied by diffusion, while most tissues demand branches of blood vessels (usually within a distance of 100–200 µm) to transport oxygen, nutrition, and waste. Vascularization has therefore been recognized as one of the major challenges in fabricating thick tissues and organs that survive and function for a long term [15,16]. For in vivo systems, solely relying on the natural process of vessel spouting may not be enough to supply the inner part of thick implanted tissues, because the sprouting of pre-existing vessels into the implanted tissue is slow (about 1 mm per week) [17], and the inner parts of implanted tissues may lose their viability in the process. Therefore, it is necessary to take measures to build vasculatures and promote vascularization. For in vitro cell culture systems, it is also well documented that dynamic perfusion combined with microfluidic channels or vascular network significantly improves the fluidic transport in cell culture [18,19,20,21].

There are two mechanisms of new blood vessel formation: angiogenesis and vasculogenesis [22]. Angiogenesis is the process whereby pre-existing capillaries sprout to form new ones. Vasculogenesis is the differentiation of precursor cells into endothelial cells and the formation of capillaries from the beginning. It is now known that both mechanisms can occur in embryonic development and after birth, so AM-based biomedical structures could utilize both of them [23]. Furthermore, for effective vascularization to support thick tissues or organs, complete hierarchical systems of the branched vascular trees and microvascular segments are believed to be indispensable [17].

In conventional scaffold-based tissue engineering, there are several important approaches to accelerate vascularization: fabricating highly porous scaffold structures that allow for the angiogenesis process; seeding endothelial cells (sometimes co-cultured with fibroblasts or other cells) that develop into vessels; and the delivery of growth factors like vascular endothelial growth factor [23,24]. AM-based techniques make it possible to have more complicated structures designed to mimic the natural vasculature networks, and to deposit specific types of cells to build an environment similar to the natural one.

### 2.2. Biologically Inspired Vascular Design

The first thing in constructing a vasculature is to learn from nature. A simple vessel could be a tubular structure with an endothelial cell seeded on the wall. To construct vascular networks, µCT and MRI can be suitably employed to obtain high-resolution 3D images of real vascular systems for specific organs [25]. When 3D images are used to generate the blueprints of AM, geometric changes in the tissue fusion process should be considered, so that corrections can be made in the blueprints [26].

There are also guidelines of designing the branching of vascular networks. For example, each vessel could have ca. three bifurcations, with a segmentation diameter ratio of ~1.3–1.6 and a length ratio of ~1.8–2.1 [27]. A more systematic description of branched vasculatures is Murray’s law, inspired by which Wu et al. have made a vascular network with a direct writing technique [20]. Their study revealed that fluidic transport efficiency is maximized when the network architecture obeys Murray’s law. An optimal hierarchical network that evolves naturally can also be found from leaf venation, as He et al. built their perfused cell-laden hydrogel structure by replicating the branches of a leaf [28].

Biologically inspired vasculature design may also be combined with analytical and computational analyses. One possible aspect is oxygen transport. By taking into account the fluidic flow, oxygen diffusion, oxygen consumption, and other factors, one could model oxygen distribution in the AM-based structure [23]. Oxygen-sensitive microelectrodes and dyes could be applied to have measurement in the real structure. A similar approach could be applied to ensure the transport of other nutrition or waste from the vascular structure. Another aspect is fluid dynamics, for which mathematical models and software are readily available, to optimize fluidic channel design in regard to shear stress and velocity distributions [29].

### 2.3. Fabricating Vascular Constructs

**Figure 1:**
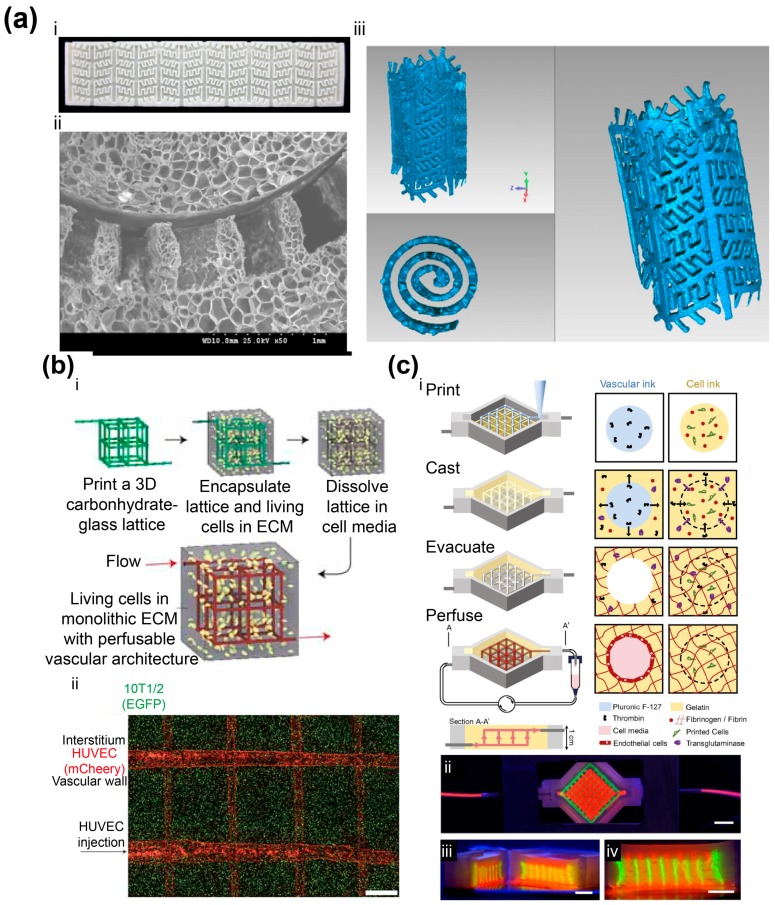
AM-based vasculatures. (**a**) 3D porous scaffold with biomimetic microchannels [39]. (i) Planar scaffold with microchannels were fabricated by replicating an AM-based mold; (ii) SEM image revealing the channel after rolling. 3D-reconstructed model of fabricated scaffold showed the interconnected microchannels. Reprinted and adapted with permission from IOP Publishing, copyright 2012; (**b**) Vascular network made with AM-based glass lattice [44]. (i) Fabrication flow of the 3D vascular network, where the glass lattice was removed after living cells were filled to form the biomedical construct; (ii) Vascularized tissue construct was fabricated by encapsulating 10T1/2 cells followed by seeding of HUVECs throughout the vascular network via a single luminal injection (scale bar: 1 mm). Reprinted and adapted with permission from Nature Publishing Group, copyright 2012; (**c**) Thick vascularized tissue [46]. (i) Schematic of the AM process. Fugitive ink was printed to form the support. ECM material was cast, followed by evacuation of the fugitive ink structure. The hollow network was then perfused for cell culture; (ii–iv) Photos of the perfusion chamber (ii) and corresponding cross-sectional views (iii,iv) (scale bars: 5 mm). Reprinted and adapted with permission from National Academy of Sciences, copyright 2016.

Researchers have explored different AM techniques for constructing vasculature structures. Nahmias et al. reported laser-guided direct writing of cells with a micrometer resolution [30]. They printed human umbilical vein endothelial cells (HUVECs) on a flat surface, after which the HUVECs self-assembled into vessels. A thin layer of gel was added on top of the cell patterns, followed by another layer of cell patterns to generate a 3D network. The endothelial viability after printing was found to be 89% ± 7%. They also demonstrated liver sinusoid-like structures, by co-culturing hepatocytes with the self-assembled HUVEC patterns. Subsequently, the observed lumens were enclosed by endothelial cells. In another report, Cui et al. developed the inkjet co-printing technique where fibrin ink was made into a scaffold with channels and human microvascular endothelial cells (HMVECs) were seeded to the inner walls of the channels [31]. Similarly, Christensen et al. employed inkjet printing to make cell-hydrogel tubular structures with horizontal and vertical bifurcations [32]. As an example of utilizing the extrusion system, Skardal et al. synthesized polyethylene glycol (PEG) derivatives mixed with fibroblasts (NIH 3T3 cells) as the microfilaments [33]. To construct the hollow tubular structures in 3D, they printed agarose microfilaments to provide the support, enclosed by the cell-containing filaments. By adjusting the concentration and flow rate of alginate and calcium chloride solution in another extrusion system, Gao et al. were able to control the crosslinking timing of hollow filaments and fuse adjust hollow filaments without the need for sacrificial support [34]. Wu et al. developed a 3D direct writing system to print fugitive ink inside hydrogel precursors [35]. The hydrogel was polymerized, and the fugitive ink was then liquefied and removed under a modest vacuum, leaving microvascular channels. To construct fluidic channels with materials that are not compatible with AM, researchers have developed a variety of techniques to utilize AM-based molds. After architectures are made with the molds, the planar biomaterial structures can be either stacked [36,37,38] or rolled [39,40] into 3D complex constructs (Figure 1a).

The capability of introducing heterogeneous cells into a 3D biomedical structure is highly desired, because the long-term viable and functional vascular constructs often require multiple cell types and proper structural arrangements. Fedorovich et al. printed hydrogels laden with cells, through which endothelial progenitor cell (EPC) tubes inside multipotent stromal cell structures were formed [41]. Norotte et al. took discrete cell spheroids as bio-ink, and the spheroids could contain different cells to form multicellular structures [42]. By using agarose rods as the support, they were able to form hollow vascular structures with branches after the agarose rods were manually pulled out in the work. Bertassoni et al. also printed agarose as sacrificial fillings to form hollow channels of vasculature [43]. In a report of Miller et al., carbohydrate glass was printed as the sacrificial template to make embedded vasculature [44] (Figure 1b). Their carbohydrate glass formula has three advantages: mechanically stiff to support the structure; dissolving rapidly in cell media; and cytocompatible. They cast cell-laden extracellular matrix (ECM) to the AM-based carbohydrate glass lattice, and the removal of the lattice left a network of hollow channels. HUVECs were then lined with the channels by injection and perfusion flow. More recently, the Lewis group developed the toolkit to co-print multiple cells, ECM, and vasculature [45]. For the fabrication of embedded vasculature, they made an aqueous fugitive ink that can be printed and removed under mild conditions. After the hollow vascular network was made inside gelatin methacrylate (GelMA), they injected human umbilical vein endothelial cell suspension into the channels, followed by perfusion cell culture. In a later report, they constructed a thick tissue (>1 cm) with embedded 3D vasculature, where the ECM contained gelatin, fibrinogen, thrombin, transglutaminase and cells [46] (Figure 1c). They also printed human mesenchymal stem cells (hMSCs) to a vascularized structure, followed by a perfusion culture beyond six weeks and the observation of hMSC development.

Due to current technical limitations of 3D biomaterial printing, it remains challenging to print microcapillaries (5–20 µm in diameter). Meyer et al. utilized two-photon polymerization that has very high resolution, and obtained branched tubular structures with inner diameter and wall thickness down to 18 µm and 3 µm, respectively [47]. Lee et al. demonstrated another possible approach based on the sprouting of vessels [48]. They first generated relatively large endothelial-cell-seeded hollow channels (0.5–1 mm in diameter) with some distance in between. Endothelial cells and supporting cells mixed with fibrin were then deposited in the space. As microcapillaries were formed from the endothelial cells in the mixture and the vascular channels sprouted out, connected vascular network at varied scales were obtained.

## 3. AM-Based Biomedical Constructs with Gradient Interfaces

### 3.1. Natural Gradient Interfaces

It is well known that transitional interfaces commonly exist between dissimilar tissues. For instance, cartilage, ligament, and muscle are connected by several interfaces, including the bone–cartilage interface and bone–ligament interface. The interfaces typically possess gradient variations in physical, biochemical, and biological properties, as well as cellular compositions [49], which may reduce stress concentrations and improve the functional performance of living tissues [50,51]. However, these interfaces are prone to injury, and available treatments often fail to restore the interfaces sufficiently, which might compromise the repair ability and the clinical outcomes.

As a typical example, the bone–cartilage interface contains the region between deep cartilage layers and the underlying subchondral bone, comprised of tidemark, calcified cartilage layer, and subchondral bone plate [52]. The bone–cartilage interface acts as a functional unit that maintains the structural integrity of the osteochondral tissue in daily life [53,54], where the subchondral bone was anchored tightly with cartilage in “comb–anchor” or interdigitations to reduce stress concentration [55]. Calcified cartilage and bone were thought to be “impermeable” [56,57,58]. However, more and more evidence indicates the presence of gomphosis and hole-like structure on native subchondral bone plate [59,60,61]. Histological imaging [55,62], scanning electron microscopy (SEM) [63,64] and dye perfusion results [65,66] confirmed the interfacial structure. The role of the interface ought to be further studied, and the structure and function of the interface have not been thoroughly explored in tissue engineering and regeneration research.

The bone–cartilage interface exhibits anisotropic structural properties. Cartilage integrates subchondral bone via 20–250 μm calcified cartilage layer (CCL), and perpendicular Collagen-II fibers (cartilage-derived) become cemented to Collagen-I fibers (bone-derived) inside the CCL [54]. The confrontal tidemark is formed through the interaction between Collagen-I and Collagen-II, and endochondral ossification (EO) as well as tidemark formation are common during skeletal development and cartilage repair. The tissue transition during cartilage repair needs to be comprehensively investigated for functional cartilage repair.

**Figure 2:**
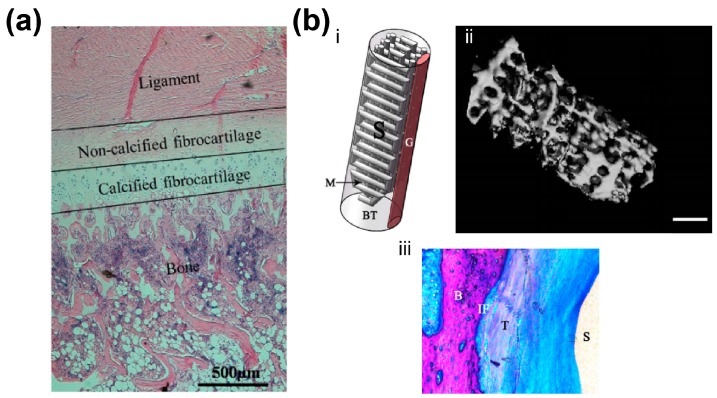

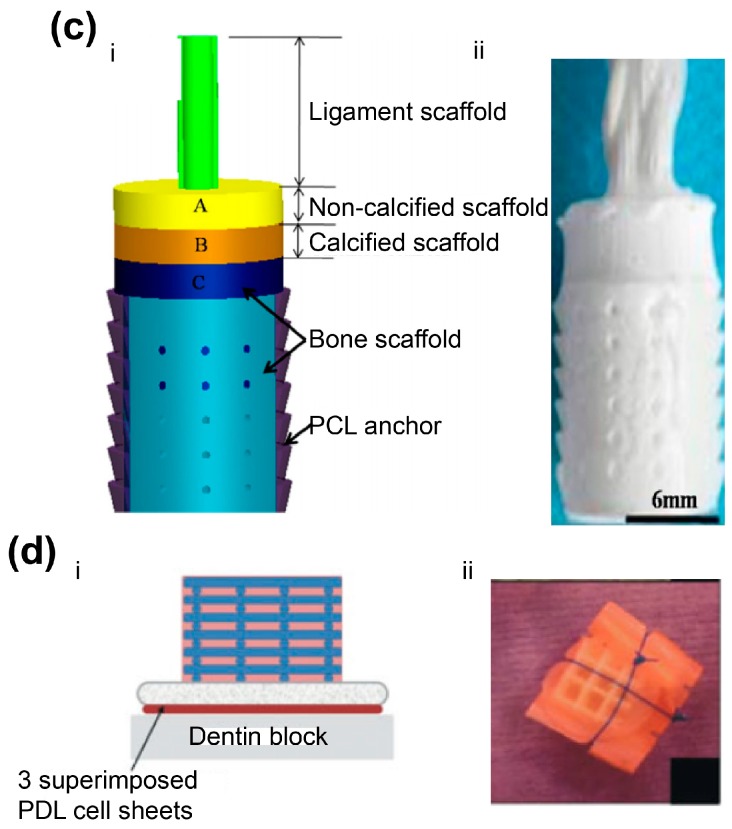
AM-based biomedical constructs with gradient interfaces. (**a**) Native ligament-to-bone interface [69]. Reprinted and adapted with permission from Elsevier, copyright 2015; (**b**) Porous PLA screw-like scaffolds coated with hydroxyapatite and their in vivo performance [70]. (i) Structure design of screw-like scaffold; (ii) New bone ingrowth in 3D scaffolds (3D reconstructed); (iii) Histology images in the rabbit ACL reconstruction, the tendon graft was in intimate contact with the new bone, with increased collagen fiber continuity at the interface part (B: Bone; IF: Interface; T: Tendon graft; S: Screw-like scaffold; Von-Gieson stain). Reprinted and adapted with permission from Nature Publication Group, copyright 2016; (**c**) Multiphased scaffolds with gradient interface [69]. (i) Schematic diagram of the scaffold design; (ii) Fabricated bone–ligament composite scaffold. Reprinted and adapted with permission from Elsevier, copyright 2015; (**d**) AM-based biphasic scaffolds with cell sheet technology to restore the periodontal interface [71]. (i) Schematic diagram of scaffold structure; (ii) Fabricated composite scaffold. Reprinted and adapted with permission from John Wiley & Sons, copyright 2013.

Similar gradient matrix heterogeneity exists at the bone-ligament interface (Figure 2a). Anatomically, the bone-ligament interface is divided into four different but continuous regions, with region-specific cell type, and matrix composition [67]. In addition, gradient matrix variation corresponds to relevant compressive stress and equilibrium modulus alteration. For example, the mechanical properties increase from the non-calcified to the calcified fibrocartilage parts is attributed to mineral presence in the mineralized fibrocartilage region [68]. Thus, the structural and compositional gradient should be considered in bone-ligament interfacial engineering, and could promote the process of bone-ligament regeneration.

### 3.2. Fabricating Constructs with Gradient Interfaces

It is well known that interface restoration remains challenging [67]. Traditionally, researchers fabricated interface structures in indirect multi-phase ways, using molding [72], electrospinning [73], or knitting [74]. However, the interfacial integrations and structures often failed to be restored by the traditional methods [75]. AM-based techniques make it possible to have complicated interface structures to be designed and more effectively restored.

Various 3D-printing techniques have been developed for constructing osteochondral gradient structure. Ding et al. reported tissue-specific, biphasic scaffolds for goat femoral head with computer-aided design and manufacturing (CAD/CAM) [76]. Polyglycolic acid/polylactic acid (PLA/PGA) scaffolds with adjusted mechanical properties were fabricated for cartilage and bone regeneration respectively, and chondrocytes and bone marrow stromal cells (BMSCs) were seeded into the scaffolds. In vivo subcutaneous implantation in athymic nude mice showed well-matched cartilage layers on the surface and stiff bone-like tissues in the bone scaffold. Cartilage and subchondral bone were well integrated with osteochondral interface regions with typical hypertrophic chondrocytes, calcified cartilage matrix, and transitional trabecular bone histological structure. AM-based molds were also applied to fabricate three- or four-layer constructs with designed interfaces to form integral osteochondral scaffolds [72,77]. However, the interfaces were not continuously formed in the structures. Dormer et al. fabricated osteochondral scaffolds with a macroscopic growth factor gradient whose delivery is spatially and temporally controlled, and in vitro results showed the engineered signal gradient was beneficial to osteochondral regeneration [78,79]. The interface integration between the chondral phase and osseous phase is crucial in engineered osteochondral scaffolds. Based on the analysis of native osteochondral histology, Bian et al. [60] applied AM and gel casting techniques to fabricate β-tricalcium phosphate/collagen scaffold with bio-inspired interface structures, with which a suitable binding force between cartilage phase and ceramic phase was achieved. Zhang et al. applied AM to fabricate a biphasic polyethylene glycol (PEG)/β-tricalcium phosphate (β-TCP) scaffold with enhanced interfacial integration. The chondral PEG hydrogel was directly cured on the ceramic interface layer by layer, and a series of interface structures were designed. The interfacial shear strength of the 30% pore area group was improved almost three times over that of the 0% pore area group, and more than fifty times over that of traditional integration (5.91 ± 0.59 kPa) [61]. The integration-enhancing interface structure design implied potential applications in osteochondral tissue engineering. Moreover, one-year implantation showed hyaline-like cartilage and tidemark formation, calcified cartilage formation, and intense subchondral bone restoration, and the creep property was restored in the 52nd week, with an equilibrium elastic modulus of 2 MPa and a permeability similar to native cartilage [80]. Cui et al. developed a 3D bioprinting technique to repair cartilage in situ, where poly(ethylene glycol) dimethacrylate (PEGDMA) mixed with chondrocytes were printed continuously for direct cartilage repair [81]. The printed cell-laden hydrogel was firmly integrated with native tissue with the presence of consistent cartilage ECM, and thus the in situ bioprinting showed potential capability of cartilage restoration.

Researchers also explored the possibility of fabricating gradient scaffolds for bone-ligament complex. Criscenti et al. utilized AM and electrospinning to fabricate bone-ligament interfaces that presented a gradient of physical and mechanical properties, mimicking the structural environment on different scales [82]. Phillips et al. developed gradient interfaces with zonal organization of osteoblastic and fibroblastic cellular phenotypes, resulting in transitional distribution of mineral deposition and mechanical properties in vitro and in vivo [51]. Liu et al. applied a desktop 3D printer to fabricate a porous PLA screw-like scaffold coated with hydroxyapatite, and subsequent in vivo results showed that the PLA/HA screw-like scaffold loaded with MSCs exhibited significant bone ingrowth and suitable bone-graft interface formation [70] (Figure 2b). Li et al. developed a novel multiphase bone-ligament scaffold of silk, tricalcium phosphate (TCP), and polyether ether ketone [83]. Their three-month in vivo results showed a robust transition between regenerated fibrous tissue and bone tunnel. He et al. presented a multi-material complex for ligament-to-bone fixation with biomimetic gradient structures including ligament region, interface region, and bone region [69]. The pull-out experiments showed early fixation stability (Figure 2c). Costa et al. [71] combined AM-based biphasic scaffolds with cell sheets to restore the periodontal interface, and in vitro study showed that periodontal attachment was restored at the dentin interface (Figure 2d).

## 4. AM-Based Multicellular Constructs

Besides complex structural organizations, most of human tissues or organs are characterized with multiple cell types. The cell–cell interactions are crucial to replicating cell-specific biological functions in vivo. For instance, hepatocytes would rapidly lose their functions once isolated from their native multicellular environment [84,85]. Although multicellular aggregates or microtissues that mimic native heterogeneous tissues can be fabricated via techniques based on di-electrophoretic forces [86] or bottom-up assembly [87], these techniques lack the ability to precisely deposit multiple cell types along with ECM-like hydrogels into a 3D micro-environment. A promising solution is tissue/organ printing that employs living cells and biologically-relevant hydrogels as the printing inks to fabricate living 3D constructs in a controlled and layer-by-layer manner. Recent advances in organ printing with multiple bioprinting heads enabled researchers to capture heterocellular organizations of native organs [88,89].

**Figure 3:**
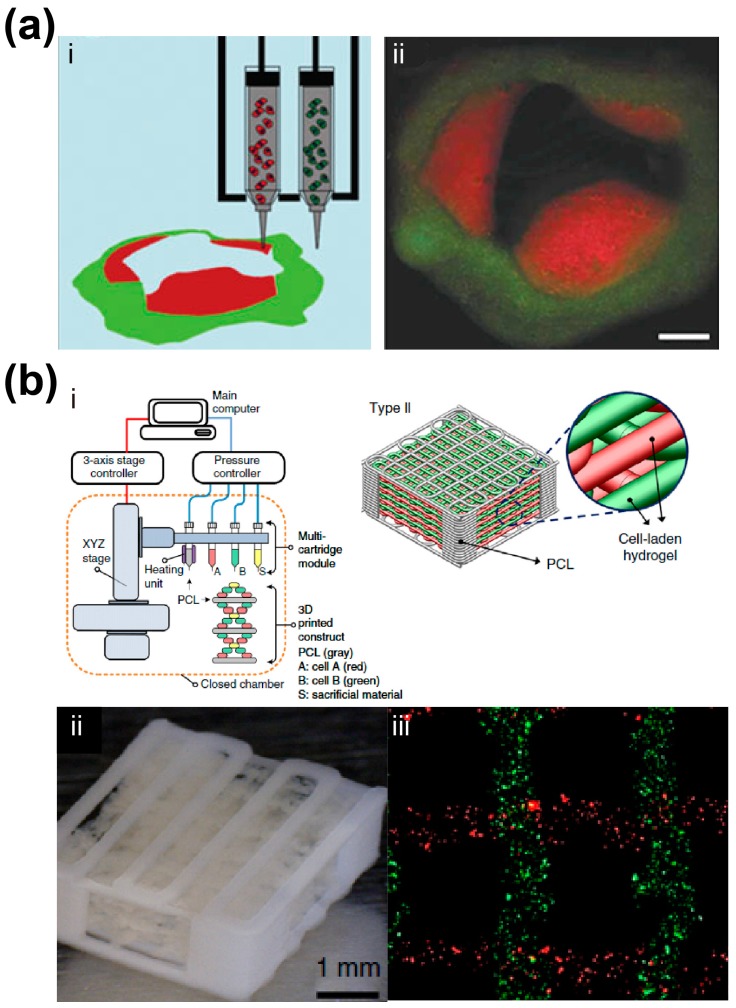
AM of multicellular tissue constructs. (**a**) AM-based aortic valve conduit [90]. (i) Schematic diagram of bioprinting process with two types of cells; (ii) Fluorescent image of the printed viable heart valve. Smooth muscle cells for valve root were labeled green, and the valve leaflet interstitial cells for valve leaflet were labeled red (scale bar: 3 mm). Reprinted and adapted with permission from John Wiley & Sons, copyright 2013; (**b**) AM-based human-scale tissues [94]. (i) Schematic of hybrid system to print human-scale tissue constructs with structural integrity and multicellular types; Photo (ii) and fluorescent image (iii) of the AM-based constructs with two types of cells. Reprinted and adapted with permission from Nature Publication Group, copyright 2016.

Duan et al. developed an extrusion-based bioprinting process that can fabricate viable alginate/gelatin heart valves with anatomical architecture and dual cell types [90] (Figure 3a). Green-labeled smooth muscle cells and red-labeled valve interstitial cells encapsulated in the printed 3D constructs remained viable during the seven-day in vitro culture period, and expressed elevated alpha-smooth muscle action and vimentin respectively. Xu et al. modified a thermal inkjet printer to fabricate complex 3D alginate constructs with three kinds of cells including human amniotic fluid-derived stem cells, canine smooth muscle cells, and bovine aortic endothelial cells, which maintained their viability, proliferation rates, phenotypic expression, and functions in vitro [91]. The printed heterogeneous constructs were found to mature into vascularized functional tissues in vivo. Gruene et al. developed a laser-assisted bioprinting process to produce 3D multicellular arrays to study cell–cell and cell–environment interactions [92]. Ma et al. recently developed a rapid 3D bioprinting process to embed human induced pluripotent stem cell derived hepatic progenitor cells (hiPSC–HPCs), endothelial cells, and adipose-derived stem cells in a hexagonal architecture for the fabrication of multicellular liver model [93]. In comparison with the liver model with pure hiPSC-HPCs, the tri-culture liver model exhibited improved liver-specific functions. These investigations have demonstrated the great potential of bioprinting to fabricate biomimetic hetero-cellular tissue constructs for various applications from regenerative medicine to in vitro tissue models. However, the main challenge associated with the existing AM strategies is the limited printing resolution which cannot precisely control the position and quantity of individual cells in the printed 3D constructs.

Another challenge for multicellular bioprinting techniques is the unsatisfactory mechanical property of the printed cell-laden hydrogels especially when the heterogeneous construct has a large scale. Multi-head or multi-arm hybrid bioprinting systems have thus been developed to simultaneously print mechanically-robust scaffold frame as well as viable hydrogel component with multicellular types [94,95,96]. Kang et al. took integrative measures to solve the challenges in several aspects and developed a 3D bioprinting system that generated human-scale multicellular constructs with anatomical geometries [94] (Figure 3b). Predefined microchannels were simultaneously incorporated into the constructs to facilitate nutrient diffusion to the surrounding cells. Clinically-relevant mandible, calvarial bone, ear cartilage, and skeletal muscle constructs have been successfully printed. With the systematic breakthrough of hybrid bioprinting, their system might evolve into a promising and innovative strategy for the scale-up and mass production of large heterogeneous tissue or organ constructs [97].

## 5. Other AM-Based Biomimetic Biomedical Constructs

The rapid development over the past decades has largely extended the capability of printing biological materials in 3D. Fabrication resolution of AM has been improved, so that smaller features now can be constructed with unprecedented precision. Nanomaterials were made into 3D-printable inks, allowing even tinier components to be placed inside the 3D structures. Such advances have been creating new possibilities of biologically inspired structural organizations and corresponding applications. Here we would like to highlight some other types of AM-based biomimetic constructs reported so far.

**Figure 4:**
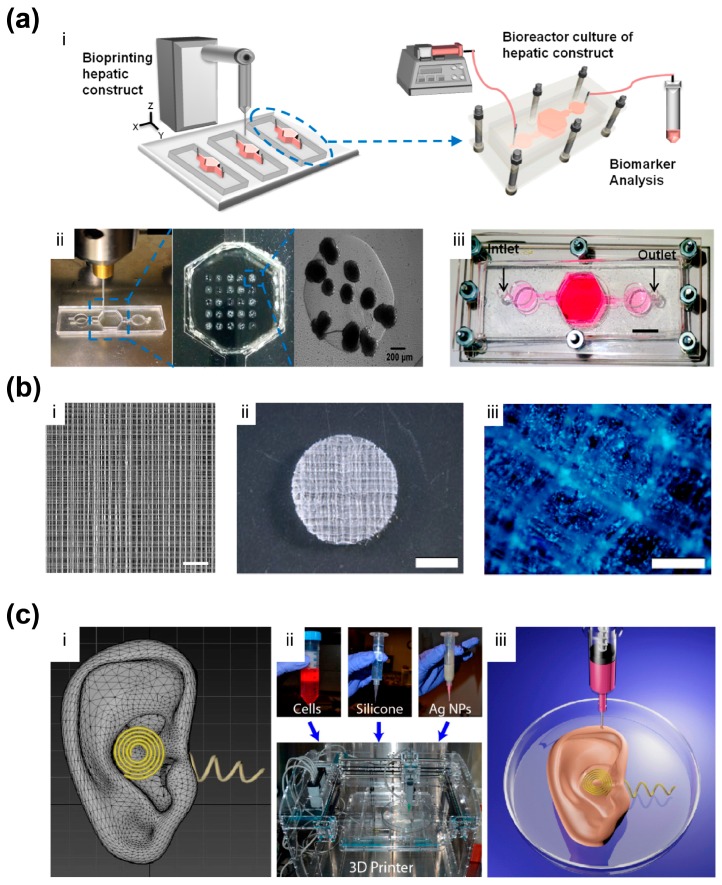
Other AM-based biomimetic biomedical constructs. (**a**) An AM-based liver-on-a-chip platform [100]. (i) Schematic of constructing the liver-on-a-chip platform; (ii) Bioprinted photocrosslinkable GelMA hydrogel-based hepatic construct within microfluidic chip as a dot array; (iii) Top view of the platform (scale bar: 1 mm); Reprinted and adapted with permission from IOP Publishing, copyright 2012; (**b**) 3D hydrogel structure reinforced by microfibers [102]. (i) Microscopic image of 3D microfiber structured was printed with PCL (scale bar: 1 mm); (ii) GelMA-HA gel reinforced with a PCL scaffold (scale bar: 2 mm); (iii) Staining confirming a homogenous distribution of the cells throughout the AM-based construct (scale bar: 200 µm) Reprinted and adapted with permission from Nature Publishing Group, copyright 2015; (**c**) AM-based bionic ear [103]. (i) CAD drawing of the AM-based ear; (ii) Photos of the AM system and corresponding inks; (iii) Illustration of the AM process. Reprinted and adapted with permission from American Chemical Society copyright 2013.

Microfluidic organs-on-chips represent an updated version of 3D cell culture systems, which utilize the microfluidic environment and stimulation mechanisms to build biological models for better physiological relevance [98]. AM technology joined these new modeling systems by constructing microscale architectures with high precision. For example, Chang et al. developed a cell-writing system, and demonstrated the possibility of printing a 3D miniaturized liver model and integrating it into a microfluidic chip for drug metabolism studies [99]. More recently, Bhise et al. printed GelMA hydrogel solution mixed with HepG2/C3A hepatic spheroids to make a liver-a-chip model [100] (Figure 4a). Bioactivity was confirmed by biomarker analysis after culture in a perfusion system for 30 days, and the response of this model to acute acetaminophen dose was found comparable to that of animal models.

3D electrohydrodynamic printing, which evolved from electrospinning, is now able to print fibers with diameters from a few micrometers to hundreds of nanometers [101]. With this printing technique, Visser et al. built AM-based microfibers as a highly organized architecture to support hydrogel [102] (Figure 4b). The mechanical properties of the construct can be controlled with the microfiber/hydrogel composition, to match native tissue (articular cartilage in the report). It was found that encapsulated human chondrocytes were more responsive to mechanical loading in terms of gene express, because this biomechanically functional structure better mimicked the in vivo mechano-environment of natural articular cartilage.

Nanomaterials possess a big variety of interesting properties and functions. By organizing such materials properly inside the AM-based constructs, one could not only mimic biomedical structures, but also biological functions. Mannoor et al. fabricated a bionic ear, where chondrocytes, hydrogel, and silver nanoparticles were AM-based to form an ear-like structure with conductive coil inside [103] (Figure 4c). Therefore, the structure had cells cultured inside and also the capability of detecting audio signals. Another way to utilize nanoscale materials and features is to regulate the interface to cells, as it has been studied in depth that nanoscale features largely affect the behavior of cells, and mimicking native biological interfaces on this scale would help to capture the biological function of cells in AM-based constructs. Castro et al. incorporated two types of nanomaterials into AM-based osteochondral scaffolds: nanocrystalline hydroxyapatite (nHA); and core-shell poly(lactic-*co*-glycolic) acid (PLGA) nanosphere that encapsulated growth factor [104]. The nHA reinforced the mechanical property of the scaffold, provided nanotexturization, and served as an osteoconductive factor. Their experiments revealed that the nHA improved cell density and spreading.

## 6. Closing Remarks and Outlook

In this review, we discussed the advances of AM-based biomimetic biomedical constructs. The ability of depositing cells and other materials in predefined locations is the key advantage of AM in biomedical studies and applications. By focusing on different biological sections, we talked about the three major directions of vasculatures, gradient interfaces, and multi-cellular systems, and highlighted some other achievements particularly on smaller scales. We believe AM for biomimetic constructs still has a lot of potential to be explored, to which technological improvements and growing knowledge will make significant contributions. Looking forward, we would like to briefly identify three prospective directions, in the hope of inspiring future research.

Four-dimensional (4D) printing stands as a new paradigm where the printed structures can respond to stimuli and even adapt to their environment over time [105]. So far, printed biomedical structures have been able to respond to temperature, water, light, etc. [106,107]. With such adaptability and additional functions, the printed biomedical structures may mimic the native organisms in a dynamic manner. However, there is ongoing debate on some important definitions and issues for this novel technology [108]. The complexity of 4D printing systems will also result in more challenges.

As we have discussed before, functional nanomaterials have been used in AM-based biomedical structures. We expect more smart components to be introduced to 3D-printing systems, especially through nanotechnologies. For example, electronics, sensors, and stimulation can be integrated, as researchers have demonstrated via non-AM approaches [109,110,111]. Such components can act as the interfaces between biological systems and machines, and AM may result in them being fabricated with more freedom and in higher efficiency.

We have discussed AM-based constructs from human scale to nanoscale, and we believe with the advancements of AM technology, biomedical constructs may be comprised of biomimetic features across multiple dimensions. As such, AM-based biomedical constructs may represent the real biological systems more faithfully and their biological activity may be further improved systematically. The research of Kang et al. has already shown the power of integrative measures to make tissue-engineered structures succeed on a larger scale and on a longer term [94].
